# Reliability and performance of commercial RNA and DNA extraction kits for FFPE tissue cores

**DOI:** 10.1371/journal.pone.0179732

**Published:** 2017-06-22

**Authors:** Palak G. Patel, Shamini Selvarajah, Karl-Philippe Guérard, John M. S. Bartlett, Jacques Lapointe, David M. Berman, John B. A. Okello, Paul C. Park

**Affiliations:** 1 Department of Pathology & Molecular Medicine, Queen’s University, Kingston, Ontario, Canada; 2 Division of Cancer Biology & Genetics, Queen’s Cancer Research Institute, Queen’s University, Kingston, Ontario, Canada; 3 Department of Surgery, Division of Urology, McGill University and the Research Institute of the McGill University Health Centre, Montreal, Quebec, Canada; 4 Diagnostic Development Program, Ontario Institute for Cancer Research (OICR), Toronto, Ontario, Canada; Universita degli Studi di Torino, ITALY

## Abstract

Cancer biomarker studies often require nucleic acid extraction from limited amounts of formalin-fixed, paraffin-embedded (FFPE) tissues, such as histologic sections or needle cores. A major challenge is low quantity and quality of extracted nucleic acids, which can limit our ability to perform genetic analyses, and have a significant influence on overall study design. This study was aimed at identifying the most reliable and reproducible method of obtaining sufficient high-quality nucleic acids from FFPE tissues. We compared the yield and quality of nucleic acids from 0.6-mm FFPE prostate tissue cores across 16 DNA and RNA extraction protocols, using 14 commercially available kits. Nucleic acid yield was determined by fluorometry, and quality was determined by spectrophotometry. All protocols yielded nucleic acids in quantities that are compatible with downstream molecular applications. However, the protocols varied widely in the quality of the extracted RNA and DNA. Four RNA and five DNA extraction protocols, including protocols from two kits for dual-extraction of RNA and DNA from the same tissue source, were prioritized for further quality assessment based on the yield and purity of their products. Specifically, their compatibility with downstream reactions was assessed using both NanoString nCounter gene expression assays and reverse-transcriptase real-time PCR for RNA, and methylation-specific PCR assays for DNA. The kit deemed most suitable for FFPE tissue was the AllPrep kit by Qiagen because of its yield, quality, and ability to purify both RNA and DNA from the same sample, which would be advantageous in biomarker studies.

## Introduction

The rapid evolution of technologies in cancer research has led to significant advances in our understanding of tumor genetics. Driven largely by high-throughput molecular technologies, there is a growing body of “omics” level data, annotated with cancer phenotypes. Such data permit the molecular profiling of an individual patient’s cancer, which is increasingly becoming more useful as disease management becomes more personalized [1]. Molecular biomarkers are emerging as a means of determining the prognoses of individual patients and predicting how individuals will respond to treatment, leading to increased research efforts in biomarker development [2].

A major consideration in biomarker development is the accrual of sample cohorts of sufficient size to permit rigorous statistical analyses during the discovery and validation phases. Currently, hospital-based pathology laboratories and biobanks are the best repositories of biospecimens linked to robust and relevant clinical and pathological information, enabling retrospective analysis of genotype-phenotype correlations. The ability to preserve morphologic and molecular information within these biospecimens allows one to use histopathological criteria, such as pathologic grade, stage, and histologic subtypes, as the basis for biomarker study design. More specifically, existing techniques for obtaining needle cores from formalin-fixed, paraffin-embedded (FFPE) tissues allow for harvesting of specific tumour grade or type, with minimal contamination by the stroma or other confounding tissue types. The promise of using archived FFPE tissues in biomarker discovery has made it more important than ever before to unlock the potential of this critical resource.

However, archived FFPE tissues present many technical challenges in molecular analysis. Formalin fixation leads to crosslinking of nucleic acids to proteins and other cellular constituents, making the extraction of these analytes difficult. In addition, age-related changes in pH can lead to the oxidation of formalin to formic acid, causing base purination and strand breaks [3]. Thus nucleic acids recovered from FFPE tissue are typically fragmented, and their performance as substrates for enzyme-based assays, such as polymerase chain reaction (PCR) and sequencing, is unreliable [4]. Furthermore, the utility of nucleic acids from FFPE tissues may also be limited by contamination with inhibitors of downstream PCR-based applications [5,6].

Establishing a reliable and reproducible method of obtaining sufficient amounts of high-quality nucleic acids from limited amounts of FFPE tissue remains a major challenge in many biomarker studies. Currently, several commercial kits are available for the extraction of RNA and DNA from FFPE tissue. While the manufacturer’s quality control process ensures a consistent performance under given experimental conditions, each of these kits has distinct performance characteristics in terms of yield and purity. In this study, we characterize and compare the performance of six RNA, six DNA, and two dual-extraction (RNA+DNA) kits using FFPE prostate cancer tissue as input samples. Furthermore, we highlight the compatibility of the RNA and DNA extracted with these kits as analytes in PCR- and hybridization-based downstream applications.

## Materials and methods

### Tissue specimens

Archived tissue was retrieved from the Department of Pathology archives of the Kingston General Hospital under the approval of the Queen’s University Health Sciences Research Ethics Board. Tumour-rich regions of interest were identified on histopathology slides and harvested from paraffin blocks using a manual tissue microarray punch (Beecher Instruments, USA). Three representative cores from each case were digitally photographed with a phase contrast microscope (EVOS FL Cell Imaging System, ThermoFisher Scientific, USA), and the mean tissue volume per core for each case was calculated based on tissue lengths and diameters measured using ImageJ [7,8]. The harvested tissue cores were further processed for nucleic acid isolation as follows.

To facilitate direct comparison between 14 commercial nucleic acid purification kits, we pooled and homogenized 120 cores from four different archived tissue blocks, totaling 37.2 mm^3^ of tissue. The pooled tissue cores were prepared for homogenization by first being deparaffinized in xylene (2 x 5 min at room temperature), washed in 100% ethanol (2 x 5 min), and then air-dried. Cores were then suspended in 150 μL of fresh 100% ethanol per mm^3^ of tissue and homogenized for 1 min at 10,000 rpm using a Power Gen Model 125 tissue homogenizer (ThermoFisher Scientific, USA).

### Nucleic acid isolation and quantification

We compared the performance of eight RNA and eight DNA extraction protocols (from 14 commercial kits) using 0.68 mm^3^ of the homogenized tissue. For each kit, the manufacturer’s protocols were followed (see Table 1 for more details). Generally, the extraction process involved rehydration of the homogenized tissue, followed by protease digestion, binding to a solid substrate, washing, and elution, with variations specific to each kit/protocol. Three technical replicates were performed for each extraction kit.

**Table 1:** DNA and RNA extraction kits compared in this study. Provided are the detailed kit names plus the names of their respective manufacturers and hyperlinks to online protocols.

Acronym	Nucleic Acid Extraction Kit	Catalogue No.	Supplier
RecAll	RecoverAll Total Nucleic Acid Isolation Kit for FFPE	1975	Thermofisher Scientific, Waltham, MA
AllPrep	AllPrep DNA/RNA FFPE Kit	80234	Qiagen, Valencia, CA
GenJet	GeneJET Genomic DNA Purification Kit	K0721	Thermofisher Scientific, Waltham, MA
PuLink	PureLink FFPE RNA Isolation Kit	K1560-02	Thermofisher Scientific, Waltham, MA
EZNRNA	E.Z.N.A. FFPE RNA Kit	R6954-01	Omega Biotek, Norcross, GA
DNeasy	DNeasy Blood & Tissue Kit	69504	Qiagen, Valencia, CA
QIAamp	QIAamp DNA FFPE Tissue Kit	56404	Qiagen, Valencia, CA
RNeasy	RNeasy FFPE Kit	73504	Qiagen, Valencia, CA
HPDNA	High Pure FFPET DNA Isolation Kit	6650767001	Roche Diagnostics, Indianapolis, IN
HPRNA	High Pure FFPET RNA Isolation Kit	4823125001	Roche Diagnostics, Indianapolis, IN
NorDNA	FFPE DNA Purification Kit	47400	Norgen Biotek Corp, Thorold, ON
NorRNA	FFPE RNA Purification Kit	25300	Norgen Biotek Corp, Thorold, ON
NucDNA	NucleoSpin DNA FFPE XS	740980	Macherey-Nagel Inc, Bethlehem, PA
NucRNA	NucleoSpin totalRNA FFPE XS	740969	Thermofisher Scientific, Waltham, MA

Two of the kits tested, RecAll (ThermoFisher Scientific) and AllPrep (Qiagen), are dual-extraction kits, which permit extraction of RNA followed by DNA from the same input tissue. For these two kits, after the homogenized tissue was treated with Proteinase K and then centrifuged, the resulting tissue pellets were used as input for DNA extraction, while the supernatant was used for RNA extraction.

For each extraction, the DNA and RNA yields were quantified on a Qubit 3.0 Fluorometer (ThermoFisher Scientific), using the dsDNA HS (High Sensitivity) and RNA BR (Broad-Range) Assay kits, respectively. The purity of the extracted nucleic acids was assessed by the A260/280 and A260/230 absorbance ratios obtained using a NanoDrop spectrophotometer.

### Inhibition assays

During nucleic acid extraction, contamination with organic compounds can inhibit the utility of the nucleic acids in many downstream molecular applications. To quantify the inhibitory effect of contaminants in the RNA and DNA extracts obtained using the different kits, we conducted inhibition assays as previously described [5,6,9]. Briefly, standard real-time PCR reactions were set up using murine genomic DNA derived from the Ep4 cell-line as the template; a primer set specific to the *HSD11β1* gene (S1 Table); and the PowerUp SYBR Green Master Mix (ThermoFisher Scientific, USA). Two microliters of either water (as a control) or extracted RNA or DNA was spiked into the above reaction mixture to yield a final reaction volume of 10 μL.

The reaction mixture was treated with uracil-DNA glycosylase (50°C, 2 min) and hot start (95°C, 2 min) steps, then cycled through denaturation (95°C, 15 sec) and annealing/extension (60°C, 1 min) steps for 40 cycles on a ViiA7 Real-Time PCR System (Thermo Fisher Scientific). Assays were performed in duplicate for each extraction kit. The cycle thresholds (Cq) across kits were then plotted using the GraphPad Prism v7 software (GraphPad Software Inc., USA). The inhibitory effect was quantified as the difference in the mean Cq between reactions spiked with the water control and reactions spiked with the extracted nucleic acid. One-way ANOVA with Bonferroni’s corrections was performed to compare for significant differences in Cq values.

### Assessment of the size distribution of RNA and DNA fragments

We postulated that the chemicals used in the various extraction kits may affect the size distribution of the final nucleic acid product. The size distribution of RNA fragments within the extracts was assessed using the RNA 6000 Pico kit on a 2100 Bioanalyzer Lab-on-a-Chip platform (Agilent Technologies, USA), and expressed as the percentage of fragments greater than 200 base pairs (DV_200_).

Endpoint reverse transcriptase PCR (RT-PCR) was also performed to assess the size distribution of RNA fragments. More specifically, it was used to determine the amplifiable fragment length of RNA extracted using five select kits (RecAll, AllPrep, RNeasy, HPRNA, and PuLink), as a means of further assessing the downstream utility of the RNA. Six PCR primer pairs were designed to span exons 2 to 4 of the human beta-2-microglobulin mRNA (RefSeq NM_004048), with expected amplicon sizes ranging from 92 to 386 base pairs (bp) in approximately 50-bp increments (S1 Table). For each RT-PCR assay, RNA extracted using select kits was converted to cDNA using the SuperScript VILO cDNA Synthesis Master Mix (Thermo Fisher Scientific) according to the manufacturer’s protocol. For each RNA sample, six endpoint PCR reactions were performed using 48 ng of template cDNA in a 20-μL reaction mixture consisting of 0.4 μM primer pair, 200 μM dNTP, 1.5 mM MgCl_2_, and 0.5 U *Taq* DNA polymerase (Thermo Fisher Scientific). The programmed profile of the PCR reaction consisted of initial denaturation at 95 ^o^C for 3 min, followed by 40 cycles of denaturation at 95 ^o^C (30 sec), denaturation at 55 ^o^C (30 sec), and extension at 72 ^o^C (1 min).

To assess amplifiable fragment length of the extracted DNA, four primer pairs were designed flanking the exon 2-intron 2 junction of the human beta-2-microglobulin gene (RefSeq NG_012920.1), with expected amplicon sizes ranging from 102 to 300 bp in approximately 65-bp increments (S1 Table). PCR amplifications were conducted as described above for each of the primer pairs, in six singleplex reactions using 100 ng of DNA extracted from the different kits as templates. Reactions containing DNA from fresh PC-3 cells (American Type Culture Collection, Manassas, USA) and double-distilled water were included as positive and negative controls, respectively. Following amplification, PCR products from each singleplex reaction were pooled for each kit, and 30 μL was run on a 3.0% Tris-borate-ethylenediaminetetraacetic acid (TBE) agarose gel at 100 V for 90 min. The gel was then stained with ethidium bromide and visualized under ultraviolet illumination using a GelDoc2000 documentation system (Bio-Rad, USA).

### NanoString mRNA assay

The NanoString platform was used to further quantify mRNA extracted from four select kits (RecAll, AllPrep, PuLink, and RNeasy) and one fresh (PC-3) cell line, whose RNA was extracted using RNeasy. Sample preparation and hybridization for the NanoString mRNA assay were performed according to the manufacturer's instructions. Briefly, 100 ng of input RNA was hybridized to NanoString 48-plex Customer Assay Evaluation (CAE) probes at 65°C for 20 hr. The solution-phase hybridization products were then processed on the nCounter Preparation Station for automated removal of excess probe and immobilization of probe-transcript complexes on a streptavidin-coated cartridge. Barcoded signals were acquired using an nCounter^TM^ DigitalAnalyzer from NanoString Technologies [10].

Data were analyzed using the nCounter™ digital analyzer software Version 2.1.1. Raw data were normalized to the geometric mean of spiked-in exogenous positive controls (to correct for differences resulting from assay efficiency such as hybridization, purification, binding, etc.), and then by subtracting the hybridization background [11,12]. The hybridization background is defined as all signals from the spiked-in negative controls that were below the mean background plus 2 standard deviations (SDs). Graphical analysis and one-way ANOVA with Bonferroni corrections were performed to assess for significant differences in mean mRNA counts between the four select RNA kits.

### RT-qPCR mRNA assays

Like the NanoString mRNA assays, RT-qPCR mRNA assays were used to further quantify mRNA extracted from the four prioritized RNA kits (RecAll, AllPrep, PuLink, and RNeasy). One hundred nanograms of RNA from each kit was converted to cDNA using SuperScript VILO mastermix (Thermo Fisher Scientific). Three housekeeping genes (*PGK1*, *KRT8*, and *HPRT1)* were quantified by TaqMan-based RT-qPCR gene-expression assay kits (Thermo Fisher Scientific) using the ViiA7 qPCR thermocycler. Statistical analyses, including graphics and multiple comparisons with Bonferroni corrections, the latter using two-way ANOVA, were performed to assess for significant differences in mean Cq values between different kits across the three genes.

### Methylation-specific PCR DNA assay

To assess the compatibility of DNA extracted using five prioritized protocols (AllPrep, QIAamp, RecAll, DNeasy, and GenJet) with enzyme-based downstream reactions, we performed methylation-specific PCR (MS-PCR), which is frequently employed in biomarker studies [13]. As preparation for MS-PCR, 100 ng of genomic DNA extracts was treated with sodium bisulfite, which converts unmethylated cytosine into uracil, and then column-purified according to the manufacturer’s protocol (EpiMark bisulfite conversion kit; NEB, USA).

MS-PCR assays were carried out as published in our previous study [14]. Briefly, 2 μL of the purified DNA was used in a 10-μL MS-PCR reaction involving amplification of targets in the bisulfite-converted CpG islands of the genes *GSTP1*, *ABCB1* and *RASSF1*, all known to be hypermethylated in prostate cancer. *Alu* repeat elements were used as the positive control [14–16]. Multiple comparisons were performed using two-way ANOVA to assess significant differences in mean Cq values between different kits for each MS-PCR gene target.

### Reproducibility of the AllPrep protocols

The reproducibility of the AllPrep extraction protocols was tested across three independent laboratories based on serial extractions of RNA and DNA from 12 FFPE prostate cancer samples. Briefly, nine cores were harvested from each of the 12 samples using a 0.6-mm Estigen Punch Set (Estigen, Estonia). To ensure uniformity in the quantity and quality of the input tissue, the harvested cores were pooled and homogenized. They were then distributed in equal-volume aliquots to participating laboratories, where RNA and DNA were extracted using the AllPrep protocols and assessed for yield and compatibility with downstream molecular applications (NanoString and MS-PCR) using the methods described above [14]. Inter-laboratory variability was determined by one-way ANOVA.

## Results and discussion

In this study, we compared the nucleic acid yield and quality from FFPE tissue cores across six DNA and six RNA extraction kits, as well as two dual-extraction kits (DNA/RNA) (Table 1). Based on their nucleic acid yield and purity, four RNA and five DNA extraction protocols were prioritized, and their utility in downstream molecular applications was further assessed using NanoString and reverse-transcriptase quantitative PCR for RNA, and methylation-specific PCR assays for DNA. Of the dual-extraction kits (AllPrep and RecAll), Qiagen’s AllPrep kit was selected as the optimal one for assessing the reproducibility of extraction protocols across three independent research laboratories.

### Nucleic acid yield and purity

The two dual-nucleic acid (RNA/DNA) extraction kits, AllPrep and RecAll, yielded 2,512.08 and 2,249.95 ng of RNA per mm^3^ tissue, respectively. The two RNA-only kits with the highest yields were the PuLink (3,603.48 ng/mm^3^) and RNeasy (2,713.04 ng/mm^3^) kits, followed by HPRNA, EZNRNA, NorRNA, and NucRNA.

For DNA, AllPrep and RecAll yielded 757.20 and 767.65 ng/mm^3^ tissue, respectively. Three dedicated DNA kits produced DNA yields comparable to those of AllPrep and RecAll: DNeasy (1236.03 ng/mm^3^), QIAamp (980.00 ng/mm^3^), and GenJet (750.59 ng/mm^3^). The HPDNA kit produced a lower yield, followed by NorDNA and then NucDNA (see Table 2).

**Table 2:** Yield and purity of RNA and DNA extracted using various kits. All extractions were performed strictly according to the respective manufacturer’s protocols (links in Table 1). Shown are nucleic acid yields, standardized for tissue input; absorbance ratios of 260 nm/280 nm and 260 nm/230 nm; and Bioanalyzer DV_200_ (i.e., the percentage of RNA fragments between 200 and 4000 bp).

Kit	RNA				DNA		
	Yield (ng/mm3)	260/280	260/230	DV200	Yield (ng/mm3)	260/280	260/230
HPRNA	2288.89	1.91	1.55	69	-	-	-
NucRNA	985.99	1.99	1.63	10	-	-	-
PuLink	3603.48	1.92	1.67	62	-	-	-
NorRNA	1662.61	1.85	1.05	70	-	-	-
EZNRNA	1845.12	2	1.55	31	-	-	-
RNeasy	2713.04	1.97	2.11	41	-	-	-
RecAll	2249.95	1.93	1.33	43	767.65	1.84	1.61
AllPrep	2512.08	2.01	1.4	54	757.2	1.77	1.2
GenJet	-	-	-	-	750.59	1.8	1.81
QIAamp	-	-	-	-	980	1.79	1.32
DNeasy	-	-	-	-	1236.03	1.91	1.45
HPDNA	-	-	-	-	536.53	1.87	2
NorDNA	-	-	-	-	468.53	1.91	2.09
NuDNA	-	-	-	-	429.71	1.99	1.87

By spectrophotometric assessment, all of the RNA extraction kits produced A^260/280^ ratios close to 2.0, consistent with highly “pure” samples. In contrast, with the exception of the RNeasy kit, all of the RNA extraction kits produced A^260/230^ ratios that indicated significant impurities (Table 2). Similarly for DNA, the A^260/280^ ratios were near or above 1.8 for all kits. In contrast, the A^260/230^ ratios for the RecAll, AllPrep, QIAamp, and DNeasy kits indicated potential organic contaminants in the eluent (Table 2). Contaminants such as EDTA, phenol, heme, and carbohydrates all have absorbances near 230 nm [5,9,17–19]. Given that these contaminants can inhibit downstream applications [4,20], we undertook a series of assays to quantify their inhibitory effect [6,9].

### Inhibition effects on downstream applications

**Fig 1:**
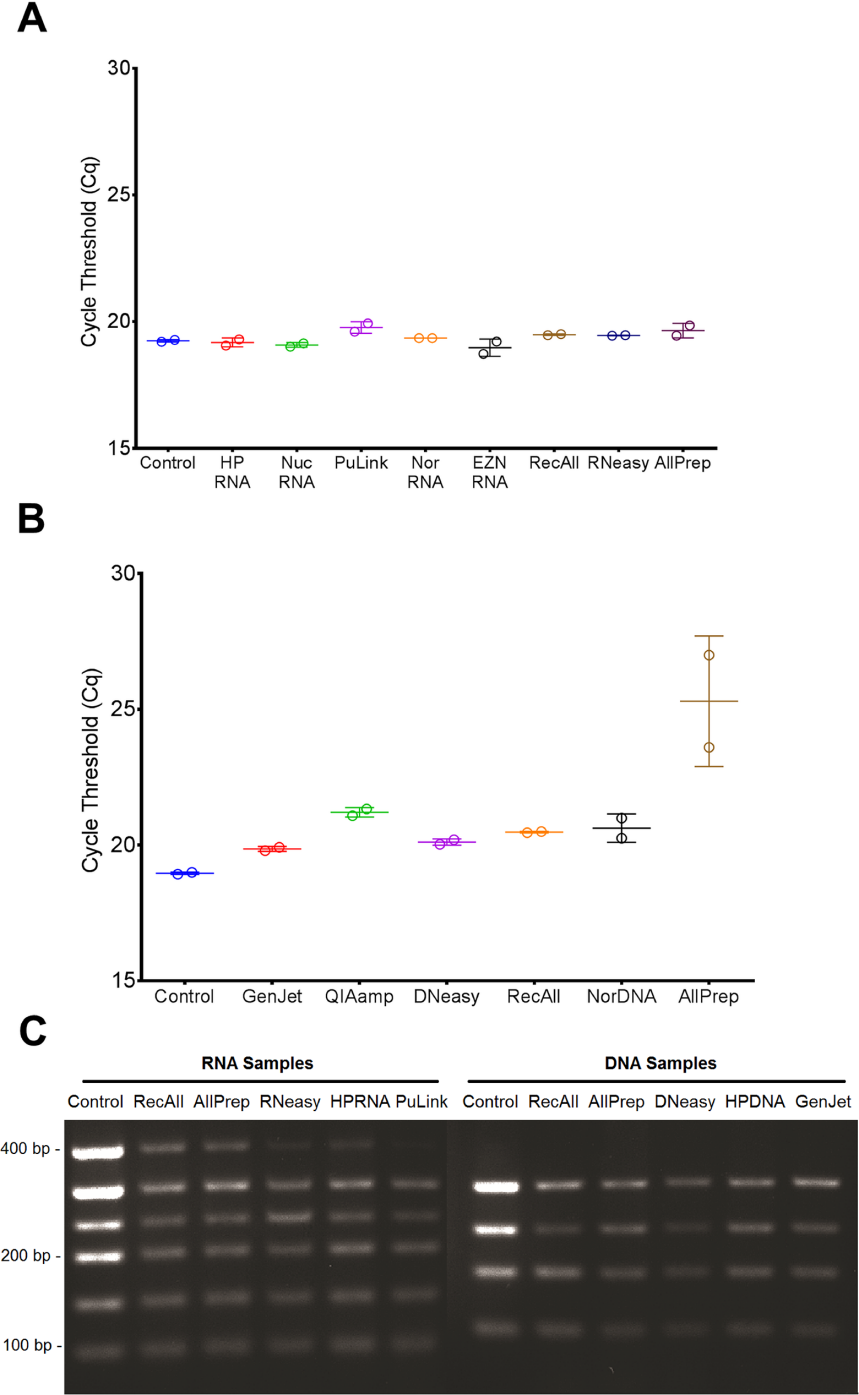
Inhibition assays. Inhibition assays were set up as qPCR reactions using murine genomic DNA; a primer set specific to the mouse-*HSD11β1* gene (S1 Table); and the PowerUp SYBR Green Master Mix. The reaction mixture was spiked with water (as a control) or extracted RNA or DNA from various kits. Shown are the Cq values for the control vs. reactions spiked with RNA (A) and DNA (B), in duplicate, with error bars representing standard deviations. (C) Fragment distribution of amplified RNA and DNA samples from select kits with corresponding positive controls (fresh PC-3 cells).

The inhibition assay was designed to quantify the impact of any inhibitors in eluted RNA and DNA samples on downstream applications. Extracted RNA and DNA were spiked into a non-target PCR reaction, and the observed delay in the Cq value of the RNA- or DNA-spiked reaction relative to that of the control (water-spiked) was interpreted as the extent of the inhibitory effect. One-way ANOVA with Bonferroni’s correction showed that none of the RNA tested had any effects on the Cq, relative to the control, indicating that the RNA was pure (Fig 1A; p > 0.05). On the other hand, a significant difference was observed between the Cq values for the AllPrep DNA-spiked reactions and the control (Fig 1B, p < 0.05). The other DNA extraction kits showed no significant differences in Cq values compared to the control reaction.

To further evaluate the inhibitory effect of the AllPrep DNA on PCR reactions, we carried out additional inhibition assays using independent prostate and breast cancer samples (see S4 Fig and S2 Table). Specifically, DNA was extracted from the respective samples using the AllPrep protocol, and then spiked into non-target PCR reactions at full concentration, at a 1:10 dilution, and at a 1:20 dilution. At full concentration, significant delays in Cq were observed for both of the prostate cancer samples, but not for the breast cancer sample. With both prostate cancer samples, the delays were reversed by a ten-fold dilution of the DNA prior to spiking.

Using the AllPrep kit on needle cores of FFPE tissue, a typical extraction yielded DNA concentrations ranging from 20 to 50 ng/μL. Thus, in our inhibition assay, the concentration of the spiked DNA in the reaction mixture would be in the range of 0.2 to 0.5 ng/μL of final reaction volume. This is well within the range of template DNA concentrations for most PCR-based applications. Thus, while the AllPrep DNA contains impurities that can inhibit PCR reactions, these are sufficiently diluted in routine use to nullify any inhibitory effect.

### Assessment of the size distribution of RNA and DNA fragments

Practical considerations in biomarker development include ease of specimen procurement, biomarker stability under archived conditions, and compatibility of the assay with established clinical workflow. Currently, FFPE tissues are the standard specimens for diagnosis and represent a vast repository of research specimens linked with long-term clinical follow-up data. Although past studies have demonstrated utility for nucleic acids extracted from archived specimens in genomic analyses [21], it is well documented that their degradation into small fragments pose technical challenges for molecular methods [4]. Formalin-fixation leads to the formation of crosslinks which increase sensitivity of the strands to mechanical stress and decrease accessibility of polymerases and other enzymes [22].

We postulated that the chemicals employed in the various extraction kits may affect the size distribution of the final nucleic acid product. We evaluated the size distribution of RNA products using the Bioanalyzer platform. Given that many downstream methods used for genotyping and Next Generation Sequencing are designed for templates > 150 bp, we quantified the amount of nucleic acid fragments > 200 bp as the percentage corrected area under the electropherogram. We found a wide range of DV_200_ values from 70% (NorRNA) to 10% (NucRNA). Notably, AllPrep and RecAll, the two kits that permit dual extraction of DNA and RNA, had DV_200_ values of 54% and 43%, respectively (Table 2). Among RNA-only extraction kits, RNeasy extracts yielded the largest fragment distribution peak in the electropherogram (S1 Fig). It is important to note that DV_200_ values represent relative, rather than absolute, amounts of fragments > 200 bp and thus do not necessarily reflect the performance of nucleic acids for use in downstream reactions such as PCR.

To assess the compatibility of the nucleic acids in PCR reactions more directly, we performed a series of endpoint PCR assays. As expected, RNA and DNA templates prepared from cultured cells yielded considerably stronger bands compared to templates prepared from equal amounts of RNA or DNA extracted from FFPE tissues (Fig 1C). Products from all five RNA protocols produced visible bands at 92, 142, 200, 248, and 303 bp, with the RecAll, AllPrep, and HPRNA products showing the highest band intensities. At 386 bp, only the RNA extracted using the RecAll and AllPrep kits produced appreciable bands (Fig 1C). For the five DNA protocols, discernible bands were seen at all amplicon sizes (102, 165, 225, and 300 bp), with slightly weaker intensities for DNeasy (Fig 1C).

The utility of end-point PCR-based assays for assessing the quality of RNA and DNA from FFPE tissue has been demonstrated previously [23,24]. In these assays, the relative intensities of the various sized bands for a given sample reflect the size distribution for that sample, while the differences in the band intensities between the samples for a given primer pair reflect the varying extent of contamination. In the current study, no clear correlation was observed in the fragment size distribution as determined by end-point PCR versus Bioanalyzer analysis. It is noteworthy, however, that extensive fragmentation of both RNA and DNA, which is typical for FFPE samples, made the interpretation of the Bioanalyzer electropherogram unreliable (S1 Fig).

Based on yield and purity, five DNA protocols (AllPrep, RecAll, DNeasy, QIAamp, and GenJet) and four RNA protocols (AllPrep, RecAll, PuLink, and RNeasy) were prioritised for further quality assessment. Both dual-extraction kits (AllPrep and RecAll) were included based on their capacity to extract both RNA and DNA from the same tissue source, which provides significant advantage in biomarker studies. The prioritised kits were further evaluated using NanoString, RT-qPCR expression, and MS-PCR assays.

### mRNA assessment by NanoString and RT-qPCR

The NanoString platform has previously been used for direct, digital quantitation of specific mRNA transcripts through hybridization to two sequence-specific, color-coded probes [12]. As this technology is strictly hybridization-based, it avoids the use of reverse transcription and amplification, and thereby eliminates potential amplification bias common to PCR. The RNA levels determined using NanoString are likely to be more accurate since this assay allows direct detection with molecular barcodes. NanoString-based systems are sensitive, reproducible, and highly multiplexable for detecting nucleic acid targets across all levels of biological expression levels [10,25], and are becoming increasingly common in biomarker studies.

**Fig 2:**
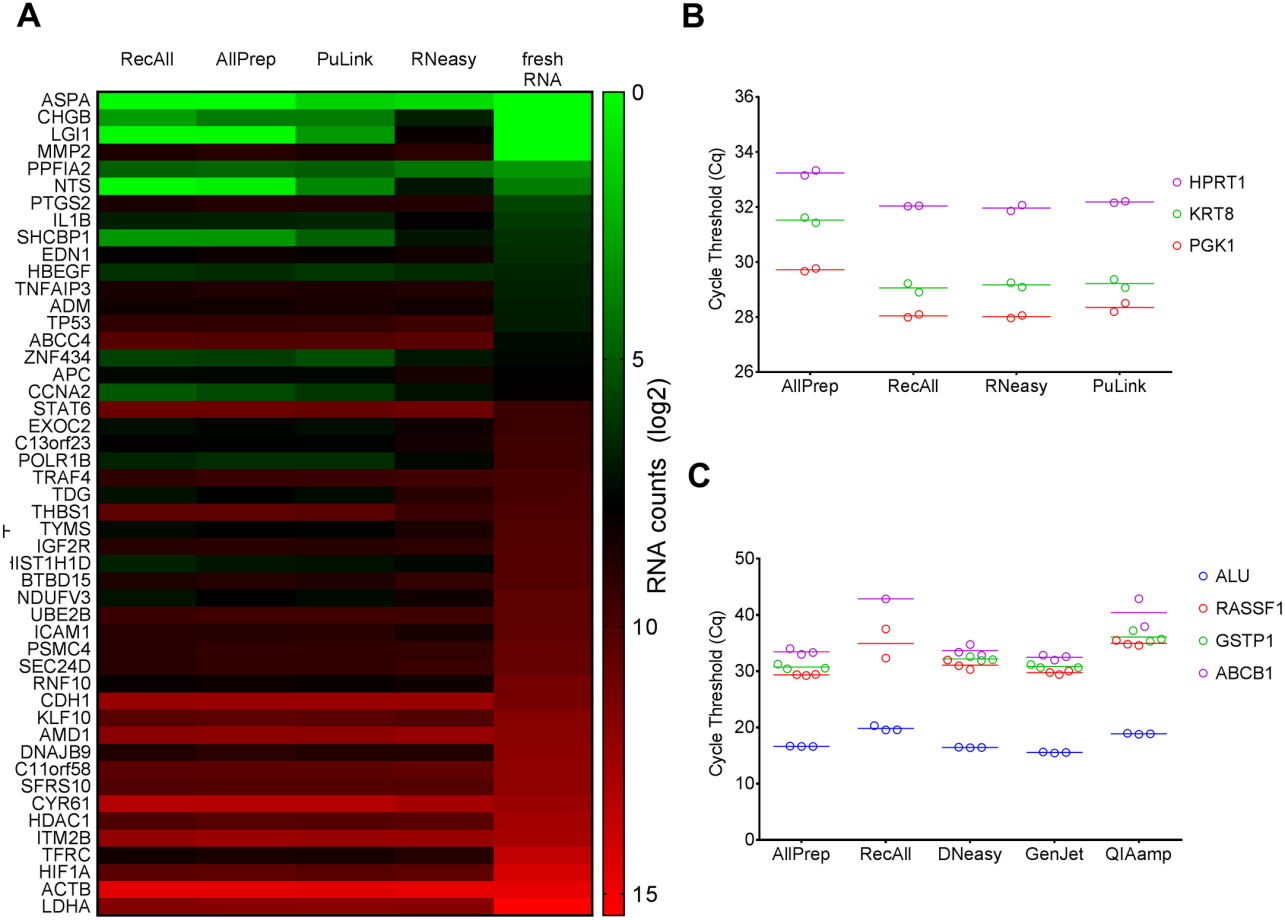
Assessments of the suitability of RNA and DNA extracted from prioritized kits for typical down-stream applications. (A) NanoString-based nCounter results showing counts of RNA extracted from four kits plus RNA extracted from fresh PC-3 cells (using the RNeasy kit). Signal counts from each of the 48 genes are shown as heatmaps (in log_2_ scale), with red and green representing the highest and lowest number of counts, respectively. (B) Cycle threshold (Cq) values in duplicate from RT-qPCR of three housekeeping genes (*PGK1*, *KRT8*, and *HPRT1*) using RNA extracted via four kits. (C) Cq values from MS-PCR for three genes commonly methylated in prostate cancer (*RASSF1A*, *GSTP1*, and *ABCB1*) as well as *Alu* repeats (as a control) across the five prioritized DNA kits. Data points for replicates are shown, with lines indicating the median Cq values. See supplementary S2 Table for more detailed data and statistical analysis.

RNA extracted from four select kits and one fresh (PC-3) cell line were run on the NanoString platform, and the mean differences between total mRNA counts were compared by one-way ANOVA. Pairwise comparisons between RNA extracted from the cell line and each of the four selected kits showed no significant difference in total signal counts (Fig 2A and S2 Fig).

By RT-qPCR, RNA from all four prioritized RNA protocols were successfully used to amplify the three house-keeping genes tested (*PGK1*, *KRT8* and *HPRT1*), showing the overall compatibility of these protocols with downstream gene expression assays. Of these, the AllPrep protocol yielded significantly higher Cq values for all three genes (two-way ANOVA, p < 0.0001; Fig 2B). Given that the size distribution of AllPrep RNA compared favourably to those of RecAll and PuLink by both DV_200_ and end-point PCR, the higher Cq values (i.e., reduced amplification) cannot be attributed to the fragment size. Rather, the data suggest the presence of contaminants in AllPrep RNA that have an inhibitory effect on the PCR reaction, consistent with the delayed Cq also observed with AllPrep DNA in the inhibition assays.

### Methylation-specific PCR (MS-PCR)

DNA base modifications, especially methylation of cytosine in the CpG islands, play a critical role in the regulation of gene expression. MS-PCR is a robust method to detect cytosine methylation. It involves the conversion of unmethylated cytosine into uracil, which is subsequently detected by PCR amplification using primers specific to the conversion products. We performed MS-PCR on DNA extracted using the five prioritized protocols to assess whether these DNA extracts are compatible with this downstream application.

We targeted three genes known to be hypermethylated in prostate cancer, namely *GSTP1*, *ABCB1*, and *RASSF1*, along with the *Alu* repeat sequences as a positive control [16,26,27]. DNA from the AllPrep, DNeasy, and GenJet kits resulted in similar Cq values (Fig 2C). Compared to the other kits, and using equivalent amount of DNA input, RecAll and QIAamp had significantly higher Cq values for each of the three gene targets (two-way ANOVA, p < 0.05). Moreover, using RecAll-purified DNA samples, *GSTP1* was not amplified at all, and *ABCB1* and *RASSF1* showed inconsistent amplification (Fig 2C). These results indicated that AllPrep DNA was most compatible with the methylation-specific PCR protocol used in this study.

### Reproducibility of the AllPrep extraction protocol

Inter-laboratory validation of a molecular protocol or assay is performed by molecular pathology laboratories to ensure its reproducibility and accuracy, which are critical components of competent patient care [28–30]. We aimed to identify the optimal RNA and DNA extraction protocols and then determine whether they are reproducible across multiple laboratories.

No single kit uniformly outperformed the others in all of the criteria compared, including the yield, purity, and compatibility with downstream applications. It is noteworthy, however, that the two dual-extraction kits tested reduce the amount of tissue required for analysis of both nucleic acid types by half, and further obviates concerns about matching RNA and DNA samples for integrated molecular profiling. Although both kits yielded RNA and DNA in quantities comparable to those of the dedicated RNA and DNA kits, the RNA product of AllPrep was of higher purity and longer in fragment length than that of RecAll by spectrophotometric measurements and DV_200_, respectively (Table 2). Furthermore, DNA extracted using RecAll performed inconsistently in MS-PCR, thus excluding MS-PCR as a potential downstream assay for RecAll, whereas this was not the case for AllPrep.

While the data indicate that AllPrep yields RNA and DNA products with contaminants that interfere with PCR amplification at higher concentrations, this inhibitory effect is negligible at the dilutions typically used for templates in PCR reactions. Ultimately, the choice of kit for nucleic acid isolation must be based on the overall design of the biomarker study. We placed a high priority on the metrics routinely used in preanalytical quality control, such as spectrophotometric absorbance and fragment distribution, and selected Qiagen’s AllPrep DNA/RNA FFPE kit as the optimal one for assessing the reproducibility of extraction protocols between three laboratories.

**Fig 3:**
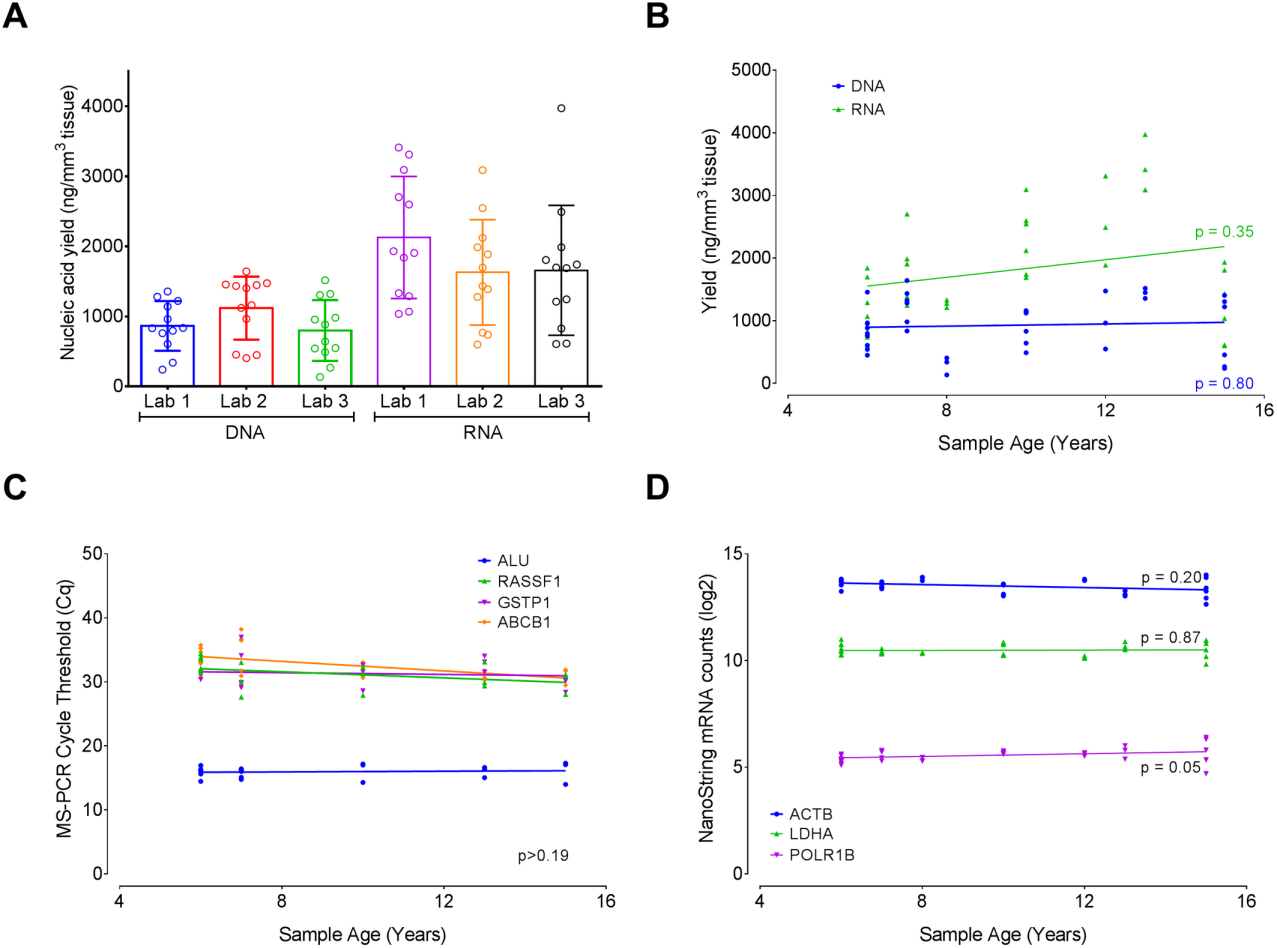
Comparison of DNA and RNA yields and quality across labs and sample age. **(**A) Bar graph (mean ± SD) comparing the yields of DNA and RNA extracted from 12 FFPE samples (circles) in three independent laboratories, using the AllPrep kit. (B) Correlation plot of DNA and RNA yield from the same 12 samples, as a function of age of sample. Each data point represents the yield for a given sample, extracted at a given laboratory, superimposed on a linear regression line. Correlation of sample age with MS-PCR amplification cycle thresholds (C) or total mRNA counts in a NanoString assay (D), based on a representative set of genes assayed in each case. Each data point represents the Cq value or the total mRNA count for a given sample, extracted at a given laboratory, superimposed on a linear regression line. Detailed data and statistical analyses are presented in the supplementary S2 Table.

One-way ANOVA analysis did not detect significant differences between laboratories in yield of DNA or RNA (p > 0.05, Fig 3A), or in the absorbance at 280, 260, and 230 nm (results not shown). The results of the MS-PCR (S3C Fig) and NanoString (S3D Fig) assays using nucleic acids extracted independently by the three laboratories were highly correlated (all Pearson’s R^2^ ≥ 0.94; p < 0.0001). Thus, the AllPrep kit produced nucleic acids that strongly correlated between the three laboratories in yield, quality, and compatibility with downstream applications. These results support a previous observation that the AllPrep kit provides reproducible protocols for the extraction of nucleic acids that are suitable for typical molecular downstream applications [14].

### Effect of sample age on yield and downstream molecular applications

The 12 FFPE samples used in the inter-laboratory study on the AllPrep kit ranged in age from 7 to 15 years. Pearson’s correlation analysis of the data failed to detect any correlation between the sample age and the yield (Fig 3B). Likewise, sample age was not significantly correlated with MS-PCR Cq values (Fig 3C) or with NanoString total mRNA counts (Fig 3D, p ≥ 0.05). While these results fail to implicate age as affecting the quality and quantity of the nucleic acids, the age of the sample as well as storage conditions are suspected to contribute to cumulative degradation of RNA and DNA through a time-dependent decrease of pH [23,31], thereby potentially negatively affecting biomarker studies. Other factors, such as fixation conditions, type of fixative, embedding procedure, and tissue type, typically remain constant for a given study.

### Limitations of this study

This study has some notable limitations. Kit performance was tested on cores but not on tissue sections, which may be better suited for certain applications. Nevertheless, cores are a popular and efficient method of selecting tissue of interest for molecular assays. In addition, the head-to-head kit comparison implemented was based on relatively recent tissue samples. Older samples were evaluated with the AllPrep kit but not with any of the other kits. Furthermore, the bulk of the data reported here derives only from prostate tissue samples. However, the AllPrep protocol yields similar results in other tissue types (S4 Fig) [14].

## Conclusions

In this study, we compared nucleic acid yield and quality across 16 DNA and RNA extraction protocols from 14 commercial kits. All the protocols tested yielded nucleic acid quantities that were compatible with downstream molecular applications, although their performances in these applications varied widely. Based on nucleic acid yield and purity, a selection of RNA (RecAll, AllPrep, PuLink, and RNeasy) and DNA (RecAll, AllPrep, QIAamp, DNeasy, and GenJet) extraction protocols were prioritized for further evaluations using methylation-specific PCR, NanoString, and reverse-transcriptase quantitative PCR assays.

The data herein provide the necessary metrics to guide the selection of a protocol that best suits the needs of the overall study design in terms of the quantity of available tissue, and the anticipated downstream applications. Overall, the AllPrep protocol reproducibly yields high quantities of matched RNA and DNA from the same tissue source. While the impurities of nucleic acids extracted using the AllPrep kit appear to impact PCR-based methods at high concentration, this effect is negligible at dilutions typical of templates in PCR-based assays. Taken together, the AllPrep kit was our preferred method for preparing nucleic acids for downstream epigenetic and gene expression studies.

## Supporting information

S1 Fig Bioanalyzer electropherograms for RNA Extractions. (A) Electropherograms for the dual-extraction kits AllPrep and RecAll. (B) Electropherograms for RNA-only extraction kits (PuLink, RNeasy, HPRNA, EZNRNA, NorRNA, and NucRNA). See supplementary S2 Table for more detailed data and statistical analysis. FU = Fluorescence units; bp = nucleotide base-pairs.
(TIF)

S2 Fig NanoString mRNA counts across prioritized RNA kits. Distribution of the NanoString mRNA counts were plotted alongside fresh PC-3 RNA (as a control) for all 48 genes from the nCounter CEA code set. Data points from 48 genes are represented as circles with median lines and SD bars. See supplementary S2 Table for more detailed data and statistical analysis.
(TIF)

S3 Fig Comparisons of nucleic acid yield and endpoint assays across three independent labs. **(**A) DNA and (B) RNA yields across the three labs. Results of the (C) MS-PCR and (D) NanoString assays performed using serial extractions of RNA and DNA from 12 FFPE prostate cancer samples. R^2^ values are Pearson’s correlation coefficients (all p values < 0.05). See supplementary S2 Table for more detailed data and statistical analysis.
(TIF)

S4 Fig Effect of AllPrep-DNA sample dilution on PCR inhibition. **(**A) Cycle thresholds (Cq) of the control sample (water-spiked) versus two undiluted prostate cancer DNA extracts and their respective 1:10 and 1:20 dilutions. The inhibition assay demonstrates a Cq shift in reactions spiked with undiluted extracts, but a significantly reduced Cq with higher dilutions across the two prostate cancer DNA samples tested. (B) No significant inhibition was seen in one breast cancer DNA extract. See supplementary S2 Table for more detailed data and statistical analysis.
(TIF)

S1 Table Primer pair names, sequences, and resulting PCR amplicon sizes. The table shows six specific primer pairs (CP1-6) for the Homo sapiens β-2-microglobulin (*B2M*) mRNA (RefSeq NM_004048), four primer pairs for *B2M* DNA (qP1-4), and one primer pair specific for the amplification of *HSD11B1* DNA. Also included are the expected PCR amplicon sizes of the respective primer pairs.
(XLSX)

S2 Table Excel book with statistical analyses used in the assessment of DNA and RNA extraction kits. This Excel book contains data used to generate figures, and statistical analyses used in data comparisons. Each tab represents data for each figure or panel, as labeled. All statistical tests reported in this study were done using the GraphPad Prism v7 software (GraphPad Software Inc., USA).
(XLSX)
