# Supplementary figures and images for: Reliability and performance of commercial RNA and DNA extraction kits for FFPE tissue cores
Source: PLoS One. 2017 Jun 22;12(6):e0179732. doi: 10.1371/journal.pone.0179732 (PMC5480995; doi:10.1371/journal.pone.0179732)

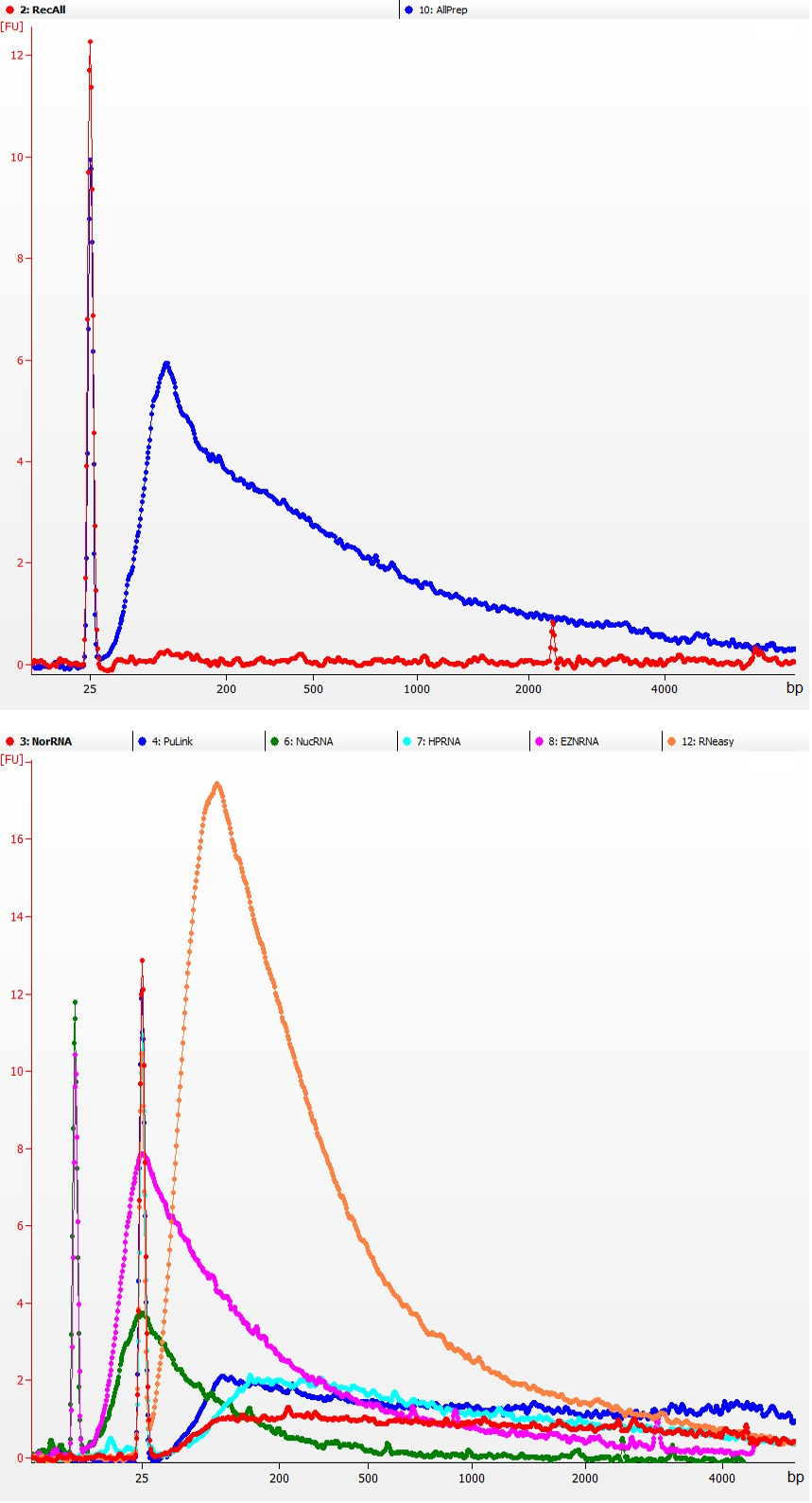

Supplement: S1 Fig — (A) Electropherograms for the dual-extraction kits AllPrep and RecAll. (B) Electropherograms for RNA-only extraction kits (PuLink, RNeasy, HPRNA, EZNRNA, NorRNA, and NucRNA). See supplementary S2 Table for more detailed data and statistical analysis. FU = Fluorescence units; bp = nucleotide base-pairs. (TIF) [file pone.0179732.s001.tif]

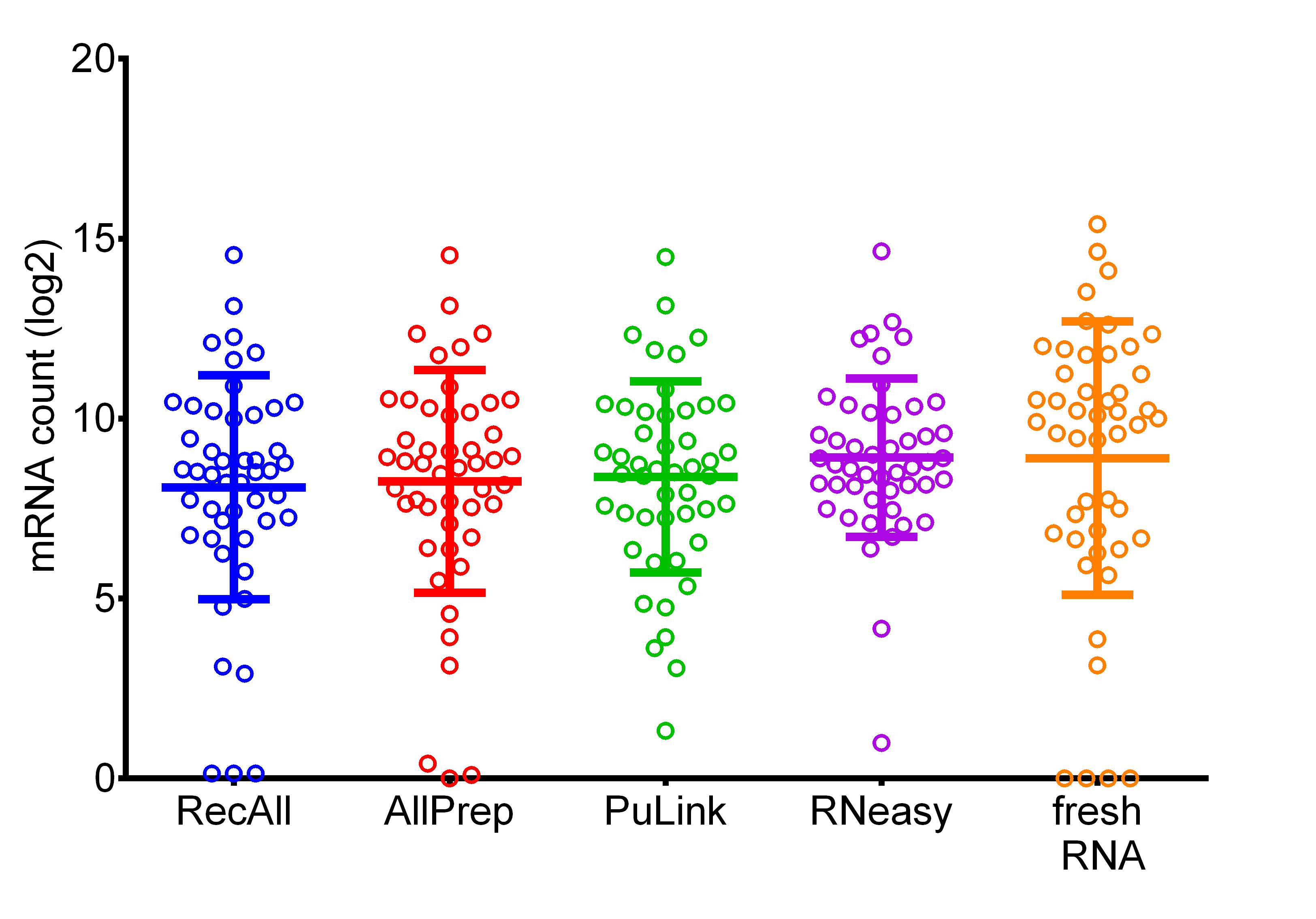

Supplement: S2 Fig — Distribution of the NanoString mRNA counts were plotted alongside fresh PC-3 RNA (as a control) for all 48 genes from the nCounter CEA code set. Data points from 48 genes are represented as circles with median lines and SD bars. See supplementary S2 Table for more detailed data and statistical analysis. (TIF) [file pone.0179732.s002.tif]

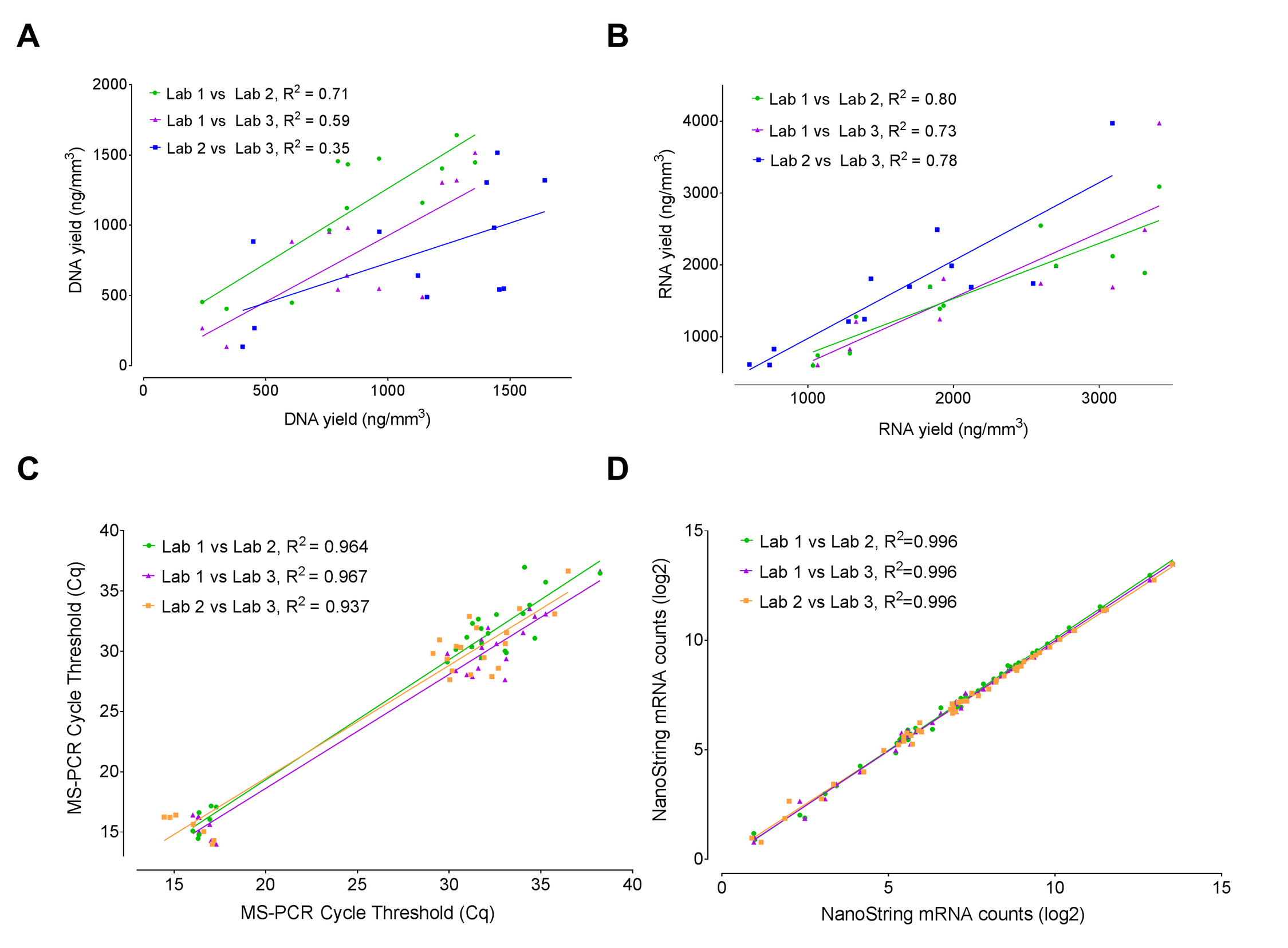

Supplement: S3 Fig — (A) DNA and (B) RNA yields across the three labs. Results of the (C) MS-PCR and (D) NanoString assays performed using serial extractions of RNA and DNA from 12 FFPE prostate cancer samples. R2 values are Pearson’s correlation coefficients (all p values < 0.05). See supplementary S2 Table for more detailed data and statistical analysis. (TIF) [file pone.0179732.s003.tif]

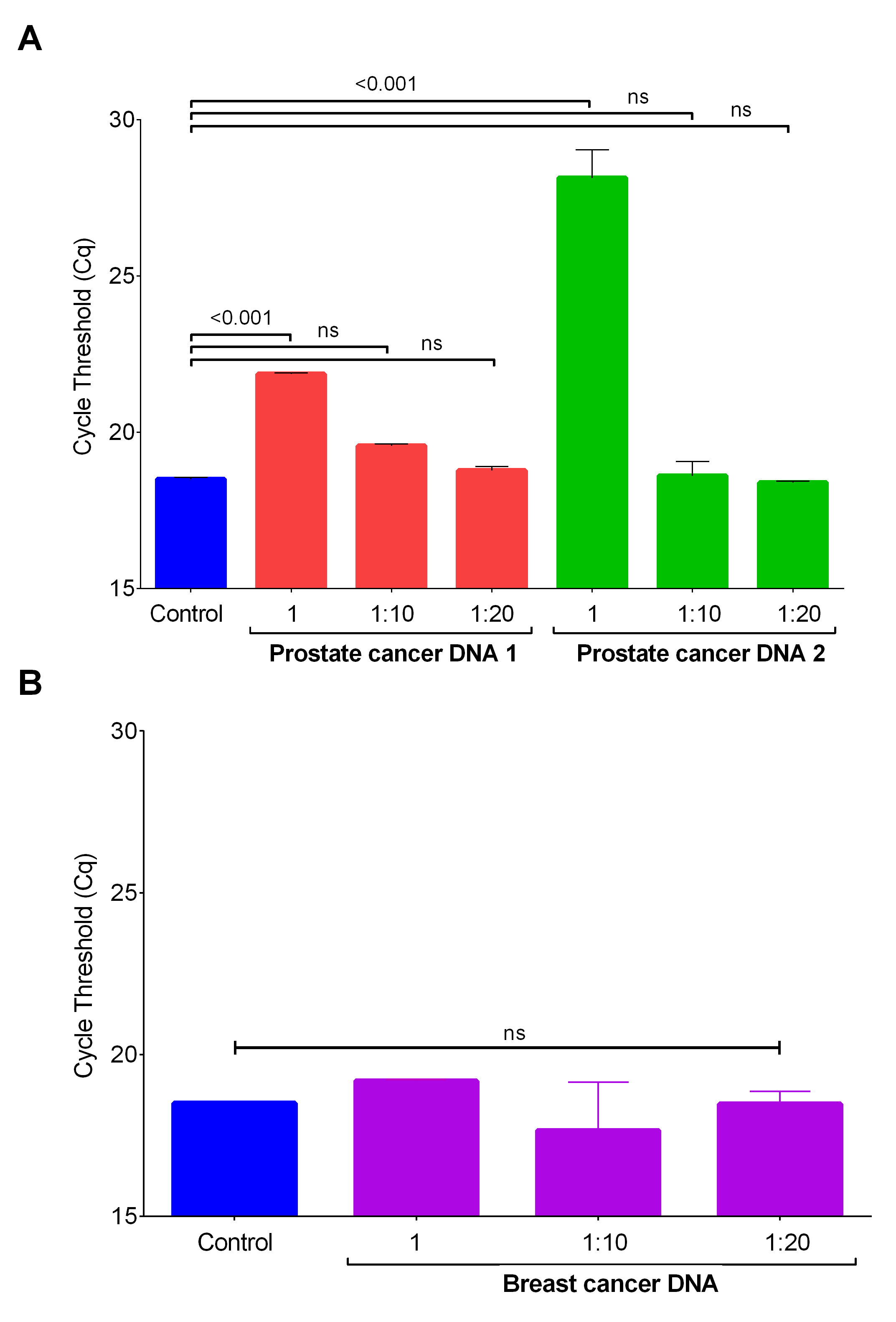

Supplement: S4 Fig — (A) Cycle thresholds (Cq) of the control sample (water-spiked) versus two undiluted prostate cancer DNA extracts and their respective 1:10 and 1:20 dilutions. The inhibition assay demonstrates a Cq shift in reactions spiked with undiluted extracts, but a significantly reduced Cq with higher dilutions across the two prostate cancer DNA samples tested. (B) No significant inhibition was seen in one breast cancer DNA extract. See supplementary S2 Table for more detailed data and statistical analysis. (TIF) [file pone.0179732.s004.tif]
